# Insulin-like growth factor binding protein-3 links obesity and breast cancer progression

**DOI:** 10.18632/oncotarget.10675

**Published:** 2016-07-18

**Authors:** Tiffany Scully, Sue M. Firth, Carolyn D. Scott, Hasanthi C. de Silva, John E. Pintar, Tailoi Chan-Ling, Stephen M. Twigg, Robert C. Baxter

**Affiliations:** ^1^ Hormones and Cancer Laboratories, Kolling Institute, University of Sydney, Royal North Shore Hospital, Sydney, New South Wales 2065, Australia; ^2^ Department of Neuroscience and Cell Biology, Rutgers University, Robert Wood Johnson Medical School, New Brunswick, NJ 08854, USA; ^3^ Department of Anatomy, Bosch Institute, University of Sydney, Sydney, New South Wales 2006, Australia; ^4^ Charles Perkins Centre, Sydney Medical School, University of Sydney, Sydney, New South Wales 2006, Australia

**Keywords:** IGFBP-3, obesity, breast cancer, BP3KO mouse, T-cell

## Abstract

Obesity is associated epidemiologically with poor breast cancer prognosis, but the mechanisms remain unclear. Since IGF binding protein-3 (IGFBP-3) influences both breast cancer growth and adipocyte maturation, it may impact on how obesity promotes breast oncogenesis. This study investigated the role of endogenous IGFBP-3 on the development of obesity and subsequently on breast tumor growth. Wild-type (WT) C57BL/6 or IGFBP-3-null (BP3KO) mice were fed a high-fat diet (HFD) or control chow-diet for 15 weeks before orthotopic injection with syngeneic EO771 murine breast cancer cells. When the largest tumor reached 1000 mm^3^, tissues and tumors were excised for analysis. Compared to WT, BP3KO mice showed significantly reduced weight gain and mammary fat pad mass (contralateral to tumor) in response to HFD, despite similar food intake. EO771 tumor weight and volume were increased by HFD and decreased by BP3KO. Despite differences in tumor size, tumors in BP3KO mice showed no differences from WT in the number of mitotically active (Ki67^+^) and apoptotic (cleaved caspase-3^+^) cells, but had greater infiltration of CD3^+^ T-cells. These data suggest that endogenous (circulating and/or stromal) IGFBP-3 is stimulatory to adipose tissue expansion and enhances mammary tumor growth in immune-competent mice, potentially by suppressing T-cell infiltration into tumors.

## INTRODUCTION

Accumulating epidemiological evidence supports the association of obesity with reduced survival from breast cancer [1]. Obesity as a risk factor for breast cancer extends across both hormone receptor-positive [2] and -negative [3] disease. However, the mechanisms by which obesity promotes breast cancer aggression remain unclear.

IGFBP-3 is the main binding partner for circulating insulin-like growth factors and regulates their bioavailability [4]. Increased levels of circulating IGFBP-3 have been associated with both increased BMI [5] and increased risk of premenopausal breast cancer [6]. High tumoral expression of IGFBP-3 has also been associated with poorer prognosis [7, 8]. IGFBP-3 can exert pro-survival or proliferative as well as pro-apoptotic effects on tumor cells [4, 9], and supports tumor growth *in vitro* by enhancing DNA damage repair and autophagy [10, 11] and by potentiating EGF receptor activation [12]. These effects appear to be tumor cell type and context-dependent. In addition to being highly expressed in some tumors, IGFBP-3 expression has also been observed in tumor endothelial cells [13] and stroma [14], possibly modulating overall tumor growth.

Mechanisms proposed to link obesity and breast cancer relate to changes that occur with the development of obesity. These include increased levels of circulating insulin/IGFs, sex hormones and cytokines released from adipose tissue [15] and the development of a tumor-supportive microenvironment [16, 17]. Since IGFBP-3 inhibits adipocyte maturation *in vitro* [18], it may influence the development of obesity. While the effects of high-fat feeding in the absence of IGFBP-3 have previously been studied [19], the consequent effects of obesity on tumor growth have not. Therefore, this study used IGFBP-3-null mice to examine the influence of endogenous IGFBP-3 on both the development of obesity in response to high-fat feeding, and on subsequent mammary tumor growth.

## RESULTS

### IGFBP-3 knock-out mice show reduced weight gain on HFD

**Figure 1:**
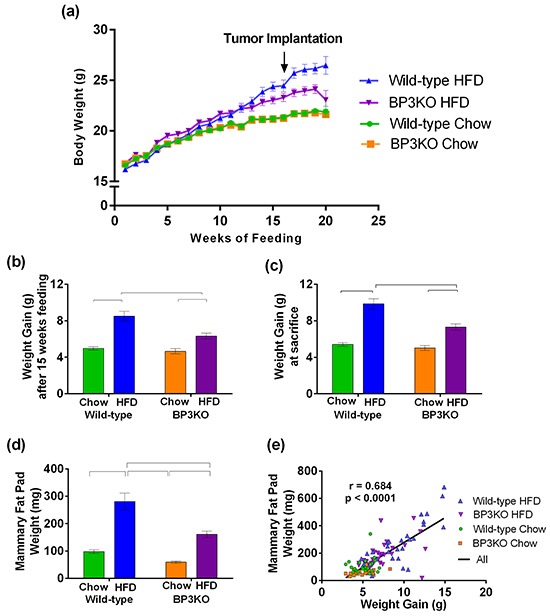
IGFBP-3 knock-out mice are resistant to diet-induced weight gain **a.** Time-course of weight gain across 15 - 20 weeks of controlled diet in female wild-type C57BL/6 and IGFBP-3 knock-out mice on chow or HFD. Mice were orthotopically implanted with tumours after 15 weeks of controlled diet (arrow). Weight gain **b.** prior to EO771 mammary tumor cell injection (p = 0.0015 for genotype, p < 0.0001 for diet, p = 0.02 for interaction) and **c.** at sacrifice (p < 0.0001 for diet, p = 0.0004 for genotype, p = 0.008 for interaction, 2-way ANOVA). n = 22 – 37 per group, data pooled from 5 experiments. **d.** Mammary fat pad weights (4^th^ right, side contralateral to tumor) (p = 0.0001 for genotype, p < 0.0001 for diet, p = 0.26 for interaction, n = 21 – 34 per group). **e.** Association of mammary fat pad weight with body weight gain. Brackets in panels (b), (c) and (d) show groups which are significantly different by *post-hoc* Tukey's test. Data are shown as means ± SEM.

Over 15 weeks of control chow diet, wild-type and BP3KO mice gained weight at equivalent rates (Figure 1a). Obesity, defined as a weight 20% above the mean for chow-fed animals, was achieved after 15 weeks of HFD (Figure 1a). Wild-type mice showed significantly greater 15-week weight gain on HFD than BP3KO mice (8.5 ± 0.5 g vs 6.3 ± 0.3 g, p < 0.05, post-hoc Tukey's test), (Figure 1b). After 15 weeks of controlled diet, 5 × 10^5^ EO771 cells were implanted into the fourth left mammary fat pad. Differences in rates of weight gain did not change after tumor implantation. At sacrifice, 21 days after tumor implantation, mice on HFD remained heavier than chow-fed mice with wild-type mice on HFD showing substantially greater weight gain than BP3KO mice on HFD (Figure 1c). Daily energy intake per mouse was similar between wild-type and BP3KO mice with control chow (44.1 ± 2.2 vs 43.0 ± 2.1 kJ) or HFD feeding (70.4 ± 8.9 vs 70.0 ± 2.4 kJ).

To determine if the reduced weight gain in response to HFD in BP3KO mice reflected decreased adipose tissue expansion, omental and mammary fat depots (contralateral to the tumor) were excised at termination, i.e. 19 - 20 weeks after HFD feeding commenced, and weighed. Depot weights between wild-type and BP3KO mice on chow diet were similar for either mammary (Figure 1d) or omental (Supplementary Figure S1a) fat. While both wild-type and knock-out mice fed HFD showed greater mammary and omental depot weights than mice on control diet, the increase in depot weight was less pronounced in knock-out mice compared to wild-type (mammary: WT-HFD vs BP3KO-HFD, p < 0.05; omental: WT-HFD vs BP3KO-HFD, p < 0.05, post-hoc Tukey's test). As such, both depots weighed significantly less in BP3KO mice than wild-type mice on HFD (Figure 1d, Supplementary Figure S1a) (mammary: 161.1 ± 11.7 vs 280.8 ± 30.7 mg; omental: 425.6 ± 44.7 vs 753.7 ± 68.5 mg, respectively). Although the absence of IGFBP-3 may influence bone growth [20], body lengths of the mice were similar between wild-type and knock-out mice (Supplementary Figure S1b), but for both genotypes, body lengths were greater overall after 15 weeks of HFD compared with their respective chow-fed controls (p<0.002, 2-way ANOVA). Total body weight gain was positively associated with mammary fat pad weight (Figure 1e). These findings suggest that the extent of fat depot expansion might explain the differences observed in total body weight gain between WT and BP3KO mice on either diet.

IGFBP-3 is the major transporter of IGFs in the adult circulation [4]. Total serum IGF-1 levels, determined post-mortem, were not significantly different between wild-type and BP3KO mice on either diet (Table 1). Total circulating IGFBP-3 levels were similar between wild-type mice on HFD and chow-diet (114.0 ± 5.7 vs 102.1 ± 7.7 ng/mL respectively, p = 0.23, *t*-test). Serum IGFBP-3 in BP3KO mice was undetectable.

**Table 1:** Circulating analyte levels in wild-type and BP3KO mice

Group					
Analyte	WT Chow	WT HFD	BP3KO chow	BP3KO HFD	Significance
Total IGF-1 (ng/mL)	72.1 ± 3.7	91.4 ± 3.6	81.4 ± 10.4	96.4 ± 16.3	G: p = 0.45 D: p = 0.07 I: p = 0.82
Total PAI-1 (pg/mL)	2096.0 ± 382.0	2121.0 ± 383.6	1637.0 ± 140	1743.0 ± 351.1	G: p = 0.22 D: p = 0.84 I: p = 0.90
Leptin (pg/mL)	429.3 ± 90.2	4892.0 ± 1132.0	692.3 ± 238.4	2063.0 ± 421.0	G: p = 0.05 D: p < 0.0001 I: p = 0.02
Resistin (pg/mL)	887.6 ± 112.4	1157.0 ± 92.3	713.0 ± 91.1	750.0 ± 32.2	G: p = 0.003 D: p = 0.092 I: p = 0.20
MCP-1 (pg/mL)	146.1 ± 40.3	158.2 ± 57.3	81.7 ± 24.5	56.4 ± 18.5	G: p = 0.05 D: p = 0.10 I: p = 0.81
Glucose (mM)	4.8 ± 0.2	4.5 ± 0.2	4.4 ± 0.2	4.8 ± 0.2	G: p = 0.98 D: p = 0.83 I: p = 0.12
Insulin (ng/mL)	0.21 ± 0.21	0.20 ± 0.05	0.44 ± 0.14	0.85 ± 0.21	G: p = 0.005 D: p = 0.16 I: p = 0.15

Circulating analyte levels were measured in wild-type and IGFBP-3 knock-out mice on chow and HFD, n = 6 − 7 per group. Data are analyzed by 2-way ANOVA (genotype and diet), for total IGF-1, PAI-1, leptin, resistin and MCP-1, glucose (n = 22 − 36) and insulin (n = 5 − 7). Variables analyzed by 2-way ANOVA are indicated by G for genotype, D for diet, and I for interaction. Statistically significant differences are underlined. Values are expressed as means ± SEM.

Among cytokines associated with obesity, circulating leptin and resistin levels were reduced in BP3KO mice compared with wild-type mice on HFD, while MCP-1 levels were similar between chow and HFD groups but were significantly lower in BP3KO than WT (Table 1). No changes in fasting glucose or total PAI-1 levels were observed between groups (Table 1). Unexpectedly, despite the significant weight gain in HFD mice of both genotypes, fasting insulin levels measured post-mortem were unchanged by HFD in wild-type mice. BP3KO mice showed significantly increased fasting insulin levels compared to WT (Table 1) (genotype p = 0.005, diet p = 0.16, 2-way ANOVA, WT HFD vs BP3KO HFD: p < 0.05, post-hoc Tukey's test, n = 5 – 7 for the subgroups analyzed for serum insulin levels).

### Characterization of the adipose tissue depots obtained from wild-type and BP3KO mice

**Figure 2:**
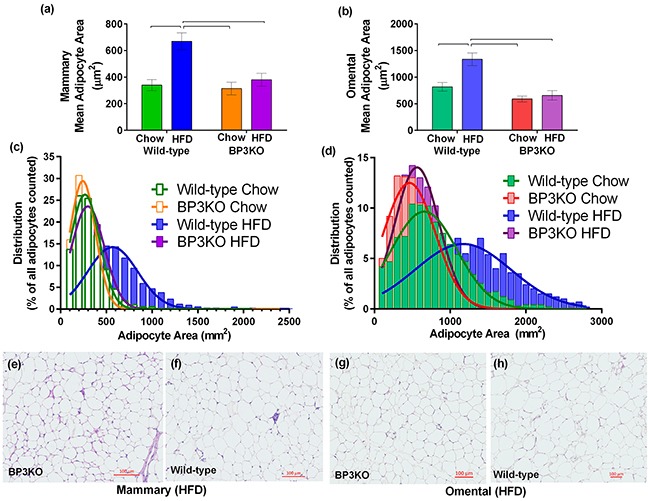
Adipocyte size in mammary and omental fat pads Adipocyte area was quantitated within the **a.** mammary, n = 6 – 7 per group, (p = 0.0062 for genotype, p = 0.001 for diet, p = 0.019 for interaction) and **b.** omental fat pads, n = 4 - 6 per group, (p = 0.0004 for genotype, p = 0.0104 for diet, p = 0.04 for interaction) of IGFBP-3 knock-out and wild-type mice on either chow or HFD. Data are mean values ± SEM; brackets over bars show groups that are significantly different by *post-hoc* Tukey's test. Adipocyte size distribution within the **c.** mammary and **d.** omental fat pads from wild-type and knock-out mice on chow and HFD. Representative images of **e, f.** mammary and **g, h.** omental fat pads from knock-out and wild-type mice on HFD are shown respectively.

Determined after 21 days of tumor growth, wild-type mice on HFD showed enlarged adipocytes in their mammary fat pads (contralateral to the tumor), with almost twice the area (669.6 ± 64.1 μm^2^) of adipocytes in either chow-fed wild-type mice (339.9 ± 41.3 μm^2^) or BP3KO on HFD (379.9 ± 48.1 μm^2^) (Figure 2a). Adipocytes in the omental fat pads of wild-type mice on HFD were also significantly larger than those in chow-fed controls and BP3KO mice on HFD (Figure 2b). In both the mammary and omental depots, fat pads of wild-type mice on HFD showed a greater frequency of large adipocytes compared to BP3KO mice on HFD (Figure 2c,d). The distribution of adipocyte size within the mammary and omental fat pads of BP3KO mice on HFD bears greater resemblance to the distribution observed in wild-type chow-fed mice, indicating that lipid accumulation within the adipocytes of BP3KO mice on HFD is greatly reduced compared to wild-type mice on HFD. Confirming our quantitative analyses in Figure 2(c,d), representative images of the mammary (Figure 2e,f) and omental (Figure 2g,h) fat pads of BP3KO mice show smaller adipocytes and therefore, a greater number of adipocytes per field of view, relative to WT mice.

**Figure 3:**
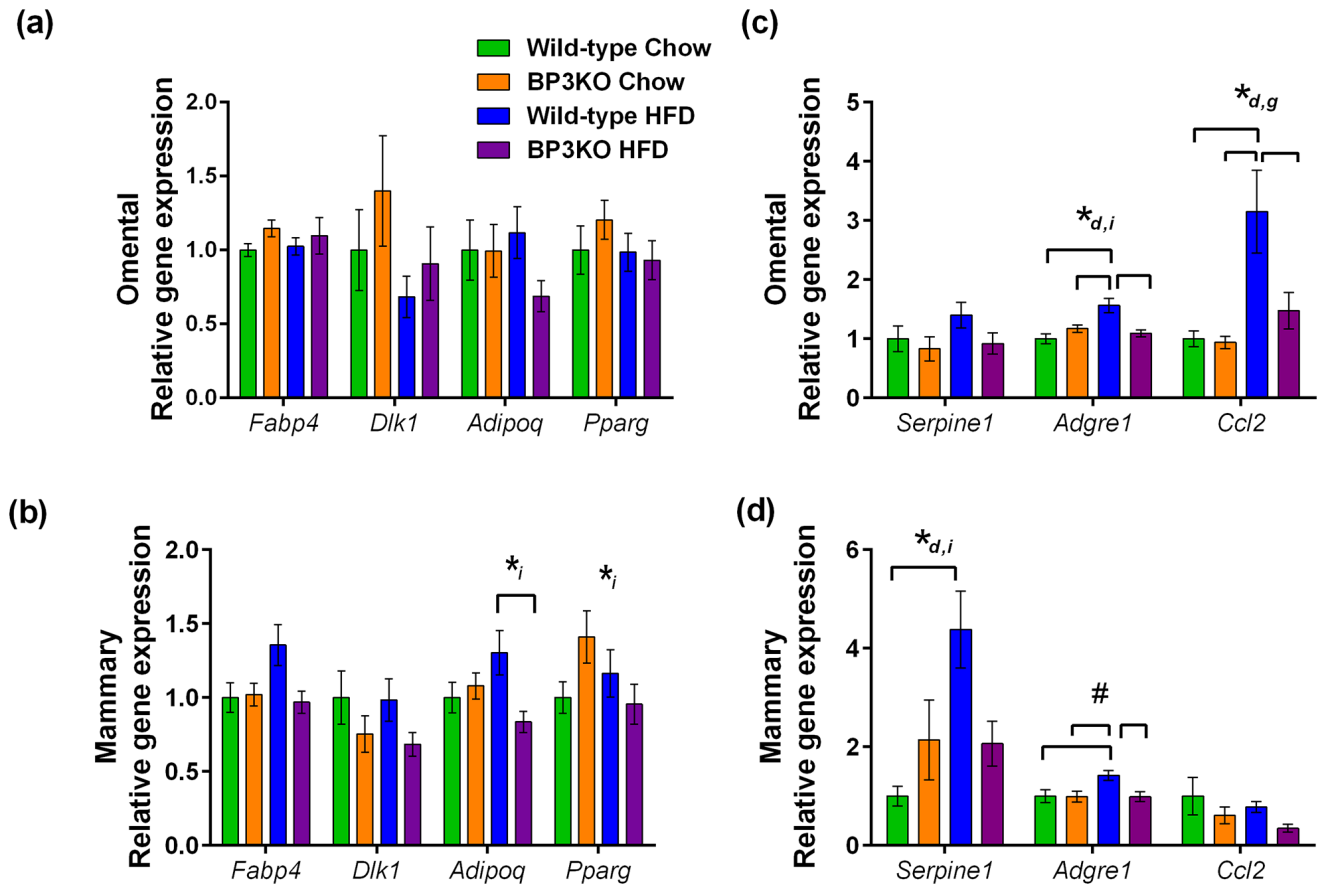
Expression of genes associated with obesity in mammary and omental fat mRNA for *Fabp4* (fabp4), *Dlk1* (pref-1), *Adipoq* (adiponectin), *Pparg* (PPARγ), *Serpine1* (PAI-1), *Adgre1* (emr1), *Ccl2* (MCP-1) was quantitated by qPCR in the **a, c.** omental and **b, d.** mammary adipose tissue depots, n = 7 – 9 per group. Genes that show statistically significant differences between groups are indicated by *^d^ p < 0.05 for diet, *^i^ p < 0.05 for interaction and, *^g^ p < 0.05 for genotype, ^#^p = 0.051 for genotype, 2-way ANOVA. Brackets over bars indicate groups that are significantly different by further testing by *post-hoc* Tukey's test.

Gene expression can differ between adipose depots in different anatomical locations [21]; therefore, both the mammary and omental fat pads were further analyzed to determine the maturation status of adipocytes within different depots. Using gene expression of fatty acid binding protein-4 (*Fabp4*) [22] and adiponectin (*Adipoq*) as markers of fully mature adipocytes, pref-1 (*Dlk1*) as a preadipocyte marker [23], and peroxisome proliferator activated receptor gamma (*Pparg*) as an indicator of adipogenesis, we found no overall difference in the omental fat pads obtained from wild-type and BP3KO mice on either chow or HFD (Figure 3a). Similarly, mammary *Dlk1* expression was unchanged among the groups (Figure 3b), but wild-type mice on HFD had higher expression of *Adipoq* (1.30± 0.15 vs 0.84 ± 0.07; p < 0.05, post-hoc Tukey's test) and trended towards higher expression of *Fabp4* (1.36 ± 0.14 vs 0.97 ± 0.07, p = 0.07 for genotype and p = 0.05 for interaction) than BP3KO mice (Figure 3b). Additionally, *Pparg* gene expression was not different between the two genotypes (Figure 3b). This suggests that in response to a high-fat diet, adipocytes in the mammary fat pad in knock-out mice are less differentiated compared with wild-type mice.

Macrophage infiltration into adipose tissue is associated with obesity [24] and may influence tumor growth. Examining macrophage infiltration by gene expression of the murine macrophage marker emr1 (*Adgre1*), wild-type mice on HFD compared to chow-fed mice showed greater infiltration into both omental (1.57 ± 0.12 vs 1.00 ± 0.09, p < 0.05, post-hoc Tukey's test) and mammary fat (1.42 ± 0.1 vs 1.00 ± 0.13, p < 0.05, post-hoc Tukey's test) (Figure 3c,d). In contrast, this expected increase in macrophage infiltration into the mammary fat depot with HFD was absent in the BP3KO mice, as *Adgre1* gene expression in both chow-fed and HFD mice were similar (0.99 ± 0.11 vs 0.99 ± 0.10, p > 0.05, post-hoc Tukey's test; Figure 3d). Furthermore, macrophage infiltration into both the omental and mammary fat of BP3KO mice was reduced compared to wild-type mice (omental: 1.09 ± 0.06 vs 1.57 ± 0.12, p < 0.05, post-hoc Tukey's test, Figure 3c; mammary: 0.99 ± 0.10 vs 1.42 ± 0.10 respectively, p < 0.05, post-hoc Tukey's test; Figure 3d).

BP3KO mice had significantly lower serum levels of MCP-1 (a chemo-attractant for macrophages, also known as CCL2) than wild-type mice (p = 0.045 by genotype, 2-way ANOVA; Table 1), but levels were unaffected by diet. MCP-1 can be secreted by a variety of cells including fibroblasts, endothelial cells, macrophages, and tumor cells [25] as well as adipocytes. To determine if the increased MCP-1 levels in the circulation of wild-type mice on HFD is related to expression within adipose tissue, gene expression of MCP-1 (*Ccl2*), within the mammary and omental depots was also measured. Whilst *Ccl2* gene expression was unaffected by HFD in mammary fat (Figure 3d), it was significantly increased by high-fat feeding in the omental fat of both wild-type and BP3KO mice (Figure 3c). This increase was less marked in BP3KO mice than wild-type. Mammary fat *Ccl2* expression in BP3KO mice trended towards decreased levels compared with wild-type mice (p = 0.073 by genotype, 2-way ANOVA) (Figure 3d).

In addition to being associated with both obesity [26] and more aggressive breast cancer progression [27], PAI-1 is also involved in the induction of senescence through the regulation of IGFBP-3 [28]. High-fat feeding increased PAI-1 *(Serpine1)* gene expression in mammary fat of wild-type mice (wild-type HFD vs chow; 4.38 ± 0.78 vs 1.00 ± 0.20, p < 0.05, post-hoc Tukey's test), but not BP3KO mice (Figure 3d), indicating that the effect of high-fat feeding on *Serpine1* gene expression in the mammary fat depot is lost in the absence of IGFBP-3.

### Lack of host-derived IGFBP-3 impairs mammary tumor growth

**Figure 4:**
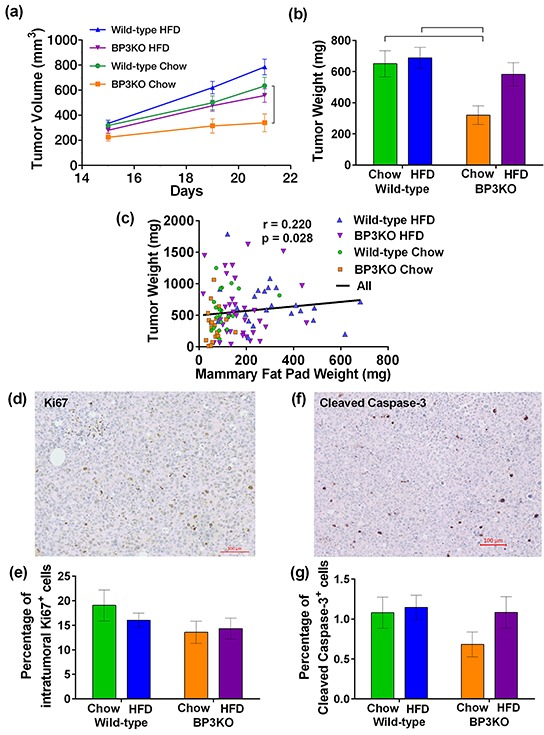
EO771 mammary tumor growth in wild-type and IGFBP-3 knock-out mice on chow and HFD **a.** Tumor volume from days 15-21 (p < 0.001 for genotype, p = 0.006 for diet, 2-way ANOVA for repeated measures). Bracket indicates significant difference by post-hoc Tukey's test. **b.** Tumor weight at sacrifice (p = 0.005 for genotype, p=0.0513 for diet, p = 0.14 for interaction, 2-way ANOVA). Brackets indicate significant differences by *post-hoc* Tukey's test. **c.** Tumor weights across all mice correlated positively with their mammary fat pad weights (significance by Spearman's correlation test). Representative images of tumors stained for **d.** Ki67 and **f.** cleaved caspase-3. Proportion of **e.** Ki67^+^ cells, (n = 12 per group, 2-way ANOVA) and **g.** cleaved caspase-3^+^ cells within the tumor (n = 11 per group, 2-way ANOVA) are not significantly different between groups. Positive cells are expressed as a percentage of the total number of cells present in the tumor. Data are shown as means ± SEM.

To examine the influence of IGFBP-3 and HFD on the growth of EO771 mammary tumors, implanted after 15 weeks of chow or high-fat feeding, tumor measurement commenced 15 days after implantation, when tumors became palpable. Tumor measurements conducted from days 15 to 21 revealed that tumors from mice on HFD had grown larger than those on chow (p = 0.0002, repeated measures 2-way ANOVA) and tumors from wild-type mice overall were larger than those from BP3KO mice (p = 0.0002, repeated measures 2-way ANOVA) (Figure 4a). Although larger tumors tended to be more necrotic and contained more fluid, the tumor volume determined *in vivo* by caliper measurement was highly associated with the post-mortem weight of the excised tumor (Supplementary Figure S2). Tumors in wild-type mice were heavier than those in BP3KO mice (p = 0.005 for genotype, 2-way ANOVA) on both chow (650.7 ± 83.1 vs 320.3 ± 59.6 mg) and HFD (688.3 ± 67.1 vs 582.5 ± 74.7 mg; Figure 4b). Mice on HFD trended towards increased tumor weights (p = 0.051 for diet, 2-way ANOVA; Figure 4b).

Compared to wild-type mice on chow, tumors from BP3KO mice on chow were significantly lighter (WT-chow vs BP3KO-chow, 650.7 ± 83.1 mg vs 320.3 ± 59.6 mg, p < 0.05, post-hoc Tukey's test). In contrast, tumors from high fat-fed BP3KO mice were not significantly different (WT-chow vs BP3KO-HFD, 650.7 ± 83.1 mg vs 582.5 ± 74.7, p > 0.05, post-hoc Tukey's test), suggesting that the decreased tumor growth associated with the absence of IGFBP-3 is not maintained in the presence of high fat-feeding. High-fat feeding may, therefore, enhance tumor growth in the absence of IGFBP-3. Tumor weight showed a weak positive association with contralateral mammary fat pad weight (Figure 4c).

### Characterization of tumor proliferation and apoptotic cell death

To further characterize tumor growth in the mice, tumors were stained for the proliferation marker, Ki67 (Figure 4d; negative control shown in Supplementary Figure S3a). The overall proportion of Ki67-positive cells within the tumors from wild-type and BP3KO mice on both diets at sacrifice was similar (Figure 4e), despite differences in tumor weights and volumes. Tumors were then stained for cleaved caspase-3 (Figure 4f; negative control in Supplementary Figure S3b), as a measure of apoptosis within the tumors. Tumors from all groups showed similar numbers of cleaved caspase-3-positive cells (Figure 4g). Therefore neither cell proliferation nor apoptosis appears to account for different tumor growth rates in response to HFD or IGFBP-3 deletion.

### Influence of IGFBP-3 on macrophage and T-cell recruitment into tumors

As elevated expression of CCL2 in tumors is associated with increased tumor growth and poorer overall survival through an increase in macrophage infiltration [29], the expression of intra-tumoral MCP-1 (*Ccl2*) and emr1 (*Adgre1*) was examined. Despite a positive association between serum MCP-1 and tumor volume (Supplementary Figure S4a) and a trend towards lower gene expression of *Ccl2* in the mammary fat depot of BP3KO mice, tumor gene expression of the macrophage marker, *Adgre1* (Supplementary Figure S4b), and *Ccl2* (Supplementary Figure S4c), was similar among the four groups, suggesting that tumor infiltration by macrophages was not affected either by HFD feeding or by IGFBP-3.

**Figure 5:**
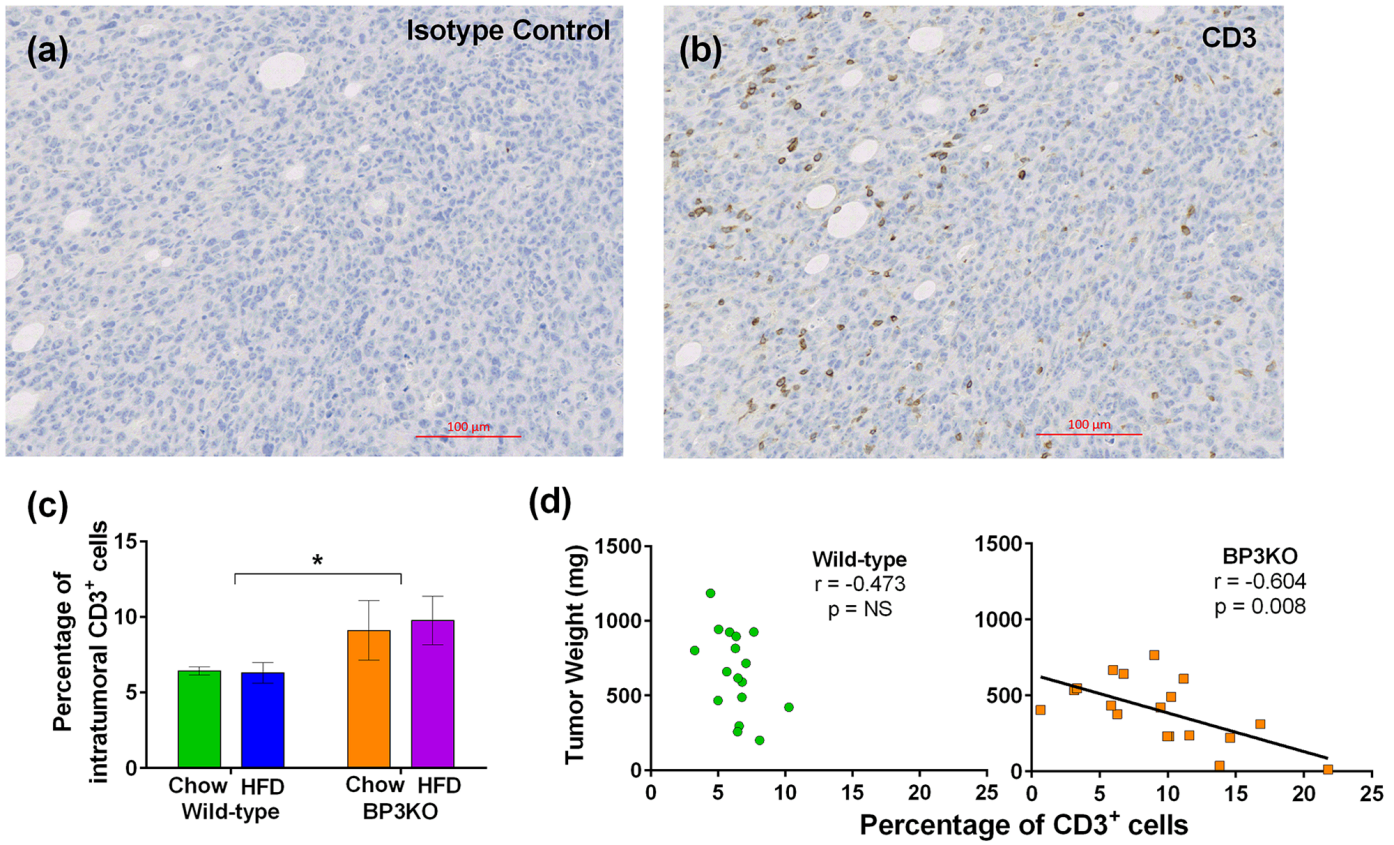
Infiltration of CD3^+^ T-cells into tumors from wild-type and IGFBP-3 knock-out mice Representative images of **a.** isotype control and **b.** CD3 staining are shown. **c.** Tumors from knock-out mice show increased infiltration of CD3^+^ T-cells, (p = 0.03 for genotype, p = 0.77 for interaction, p = 0.84 for diet n = 8 – 10 per group, 2-way ANOVA); data are mean values ± SEM. **d.** Association of CD3^+^ T-cell infiltration with tumor growth for wild-type (*left*) and BP3KO (*right*) mice, significance by Spearman's correlation test.

To gain insight into T-lymphocyte infiltration of tumors, tumors were stained for CD3, a pan T-cell marker. Figure 5 shows representative images of a positively stained tumor from a chow-fed BP3KO mouse (Figure 5b) and the corresponding isotype control (Figure 5a). Tumors from BP3KO mice showed greater infiltration of CD3^+^ T-cells (Figure 5c), but there was no apparent effect of diet. The increased intra-tumoral expression of CD3 was significantly associated with decreased tumor weight only in BP3KO mice (Figure 5d), suggesting that IGFBP-3 potentially has a novel role in suppressing T-cell infiltration into tumors.

### Tumoral blood vessel development in the absence of IGFBP-3

**Figure 6:**
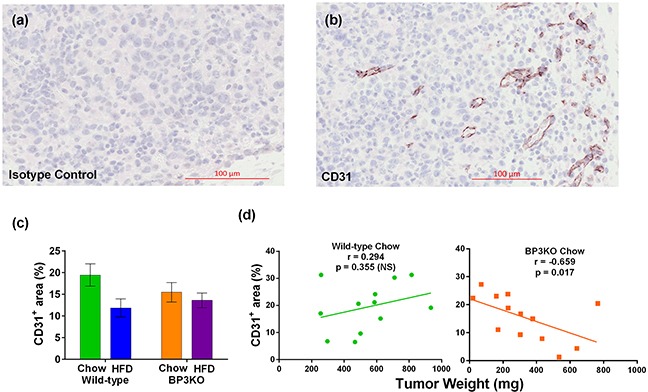
Vessel density in tumors from wild-type and IGFBP-3 knock-out mice on chow or HFD Representative images of **a.** CD31 isotype control and **b.** CD31 staining in a tumor section from a knock-out mouse are shown. **c.** Vessel densities in tumors, quantitated as number of CD31^+^ pixels as a percentage of the total tumor section area, from wild-type and knock-out mice are different by diet (p = 0.03) but not by genotype (p = 0.61), interaction (p = 0.19) (n = 13 - 14 per group, 2-way ANOVA); data are mean values ± SEM. **d.** Relationship of vessel density with tumor weight in wild-type and knock-out mice.

IGFBP-3 is involved in the formation and function [30] of blood vessels in both retinal and tumor [31] models. Tumors were examined for blood vessel development by staining for CD31 (Figure 6a,b). Vessel density in tumors, quantitated as total CD31^+^ area as a percentage of tumour section area, were similar between wild-type and BP3KO mice but were decreased overall in high fat-fed mice (p = 0.03 by diet, 2-way ANOVA) (Figure 6c). However, contrary to the weak (non-significant) positive association between vessel density and tumor weight seen in chow-fed wild-type mice, vessel density in BP3KO mice was negatively correlated with tumor weight (Figure 6d). The inverse relationship between vessel density and tumor weight in the BP3KO mice suggests that vessel formation is only stimulatory to tumor growth in the presence of IGFBP-3, supporting previous observations that IGFBP-3 has a facilitating role in the formation and function of blood vessels [30], and may thus, be linked to tumor growth.

## DISCUSSION

Since studies *in vitro* have shown effects of IGFBP-3 on both fat cell differentiation and mammary tumor cell growth, this *in vivo* study aimed to determine the influence of IGFBP-3 on the development of obesity in response to high-fat feeding and on the subsequent growth of syngeneic mammary tumors in an immune-competent mouse model. A major finding is that not only high-fat feeding, but also host-derived IGFBP-3, stimulated mammary tumor growth. This differs from the observation, in a different BP3KO model, that chemical carcinogen-induced mammary tumors grew more rapidly than in corresponding wild-type mice [32].

The diet-induced obesity in our wild-type mice, with mean body weights 20% above chow-fed controls, was not accompanied by changes in fasting glucose or insulin levels, but changes were seen in circulating cytokines associated with obesity: leptin, resistin and MCP-1. MCP-1 levels were decreased in the absence of IGFBP-3 regardless of diet, suggesting that IGFBP-3 may be stimulatory to MCP-1 synthesis and/or secretion. In comparison to other studies that have used variants of the syngeneic EO771-C57BL/6 mouse model to study the effects of obesity on breast tumor growth [33, 34], the obese phenotype induced in our study is not as pronounced. This may have been influenced by tumor growth over the final 3 weeks in our model, and by differences in the duration of feeding, the type of diet and the age or menopausal status of the mice, and may explain why the greater tumor growth in high-fat-fed compared to chow-fed mice is not as marked as previously reported [33, 34].

The development of obesity is associated with an increase in macrophage infiltration into mammary fat in both mice [16] and humans [35], perhaps related to increased levels of MCP-1. Concomitant with the unchanged circulating MCP-1 levels in the knock-out mice with high fat-feeding, macrophage infiltration into both the omental and mammary fat depots of knock-out mice in response to HFD was suppressed compared with wild-type mice. An increased number of macrophages near a developing tumor in the breast has been suggested to support tumor growth by fostering the development of a pro-angiogenic microenvironment [16]; thus decreased macrophage infiltration may be consistent with decreased tumor growth.

Mice lacking IGFBP-3 were resistant to HFD-induced weight gain – observed from week 12-13, before tumor cell implantation – and showed reduced tumor growth. Consistent with the findings of Yamada [19], where a different IGFBP-3 knock-out mouse model showed characteristics associated with the development of insulin resistance, including increased fasting insulin and glucose levels, the BP3KO mice in this study showed elevated fasting insulin levels relative to wild-type mice, that increased further with high-fat feeding. This suggests that in the absence of IGFBP-3, mice may have a greater propensity to develop insulin resistance.

The development of insulin resistance is associated with the suppression of adipose tissue development with consequent ectopic lipid accumulation [36]. While ectopic lipid deposition was not examined in this study, BP3KO mice showed significantly reduced gains in fat mass in response to a high-fat diet. A previous study [19], involving male IGFBP-3 knock-out mice on a high-fat diet, showed no differences in total body fat, although depot-specific reduction in epididymal fat mass relative to wild-type mice was seen. Our study also contrasts with that of Yakar and colleagues in which chow-fed, 8 week-old BP3KO mice had increased fat mass as a percentage of body weight [20]. In our study, chow-fed BP3KO mice showed no differences in weight gain compared to wild-type mice on the same diet, suggesting that growth rates between genotypes are similar on a chow-diet. Differences in weight gain between wild-type and BP3KO mice were only apparent after exposure to a high-fat diet. This disparity between wild-type and knock-out mice may be substantially attributable to reduced gains in fat mass, notwithstanding that all of the mice analyzed also bore mammary tumors for the final weeks of the experiment. Overall, this suggests that endogenous IGFBP-3 potentially plays a stimulatory role in the expansion of the adipose tissue depot.

Since IGFBP-3 was found to be inhibitory to adipocyte maturation *in vitro* [18], the reduced fat mass gain in HFD-fed BP3KO mice was unexpected. This discrepancy may be attributed to the complexity of adipose tissue remodelling where expansion of the adipose tissue depot involves various processes that include angiogenesis [37], extracellular matrix degradation and deposition as well as inflammation [24, 38]. IGFBP-3 can modulate both angiogenesis [30, 39] and fibrosis [40] and may, therefore, affect adipose tissue expansion independently of its influence of adipocyte maturation. Accordingly, gene expression between wild-type and knock-out omental fat pads for the preadipocyte and mature adipocyte markers, pref-1 (*Dlk1)* and *Fabp4*, as well as the adipogenesis marker, *Pparg*, was similar despite clear differences in fat pad weights.

In contrast to the lack of difference in the omental fat pad, gene expression of the mature adipocyte markers, *Fabp4* and *Adipoq* (adiponectin), in the mammary fat pads of knock-out mice were reduced compared to wild-type mice. This highlights the potential for the influence of IGFBP-3 to be depot-specific. The increased gene expression of *Serpine1* (PAI-1) associated with high-fat feeding in wild-type mice was not present in BP3KO, suggesting that IGFBP-3 may be involved in the enhancement of PAI-1 in mammary fat. Of interest, the stress-induced increment of PAI-1 *in vitro* has previously been shown to lead to increased levels of IGFBP-3 where it can act as an inducer of senescence and therefore, a potential promoter of chemo-resistance [28].

Circulating IGF-1 levels were similar in BP3KO and wild-type mice. This is consistent with another recent study employing a global deletion of IGFBP-3 in female mice [32] but contrasts with two other studies in which decreased circulating IGF-1 levels were observed in male IGFBP-3 knockout mice [19, 20]. The difference in the relationship between circulating IGF-1 and the presence of IGFBP-3 between male and female mice suggests the possibility that the compensatory effects that act to maintain IGF-1 levels in the absence of IGFBP-3 are gender-specific. The expression of other IGF binding proteins was not examined in this study, so possible compensatory IGFBP changes that might act to stabilize and transport IGF-1 in the absence of IGFBP-3 remain unknown, since circulating levels of IGFBP-2 and IGFBP-5 were unchanged in a previous study of IGFBP-3 knockout mice [32].

The reduced EO771 tumor growth observed in the knock-out mice relative to wild-type suggests that host-derived IGFBP-3 may facilitate tumor growth and/or survival, probably by an IGF-independent mechanism, since IGF-driven tumor growth is likely to be inhibited by IGFBP-3. However, the stimulatory effect of diet-induced obesity on tumor growth appeared greater in BP3KO mice, perhaps indicating that this diet-related tumor growth was driven in part by local IGF-1 bioavailability, notwithstanding the similar total circulating IGF-I levels among all groups. The stimulatory effect of IGFBP-3 on tumor growth is consistent with the clinical observation that high levels of intra-tumoral IGFBP-3 are associated with poorer prognosis [7, 41]. Aside from tumor cells themselves, components of the tumor microenvironment, such as endothelial cells [13] and fibroblasts [14], also secrete IGFBP-3 and can contribute to overall tumor levels of IGFBP-3. Since IGFBP-3 is the main circulating carrier of the IGFs, as well as a regulator of local IGF bioavailability, the absence of IGFBP-3 may affect the access of IGF-1 to cells within the tumor microenvironment. IGFBP-3 may also enhance tumor cell proliferation independent of its IGF-binding capacity, for example in response to EGF receptor signaling [42] by increasing sphingosine kinase activity [12].

It is important to reiterate that the influence of IGFBP-3 on tumor cell behaviour has been reported to be both cell-type and context-dependent, involving several signaling pathways [43]. These include growth stimulatory pathways such as EGFR signaling [4, 44] and pro-apoptotic pathways [45, 46] which may be caspase-dependent [47, 48]. In addition, IGFBP-3 has been shown *in vitro* to support breast cancer cell survival in response to hypoxia, glucose starvation and DNA-damaging agents [10, 11]. As an example of the complexity underlying IGFBP-3 activity, the IGFBP-3-associated enhancement of EGFR signalling has been demonstrated to be dependent on the type of matrix protein present, such that breast cancer cells cultured on laminin in the presence of EGF show reduced growth in response to IGFBP-3 whereas, when cultured on fibronectin, their growth is stimulated by IGFBP-3 [49]. IGFBP-3 binding to the extracellular matrix can lead to increased mammary epithelial cell attachment [50] and can also have a pro-survival effect on tumor cells exposed to lethal doses of ceramide in the presence of fibronectin [9]. Of interest, a recent study involving the relationship between obesity-related changes in the extracellular matrix of mammary adipose tissue and breast tumorigenesis has shown that in comparison to lean mice, mammary tissue-derived extracellular matrix from obese mice contained increased levels of fibronectin and increased the proliferation of premalignant mammary epithelial cells [17].

Our finding that tumoral vessel density is decreased in high fat-fed mice differs from previous observations commonly reported in the literature [34, 51]. It is important to note that our model in which mice were implanted with tumors at 22 weeks of age is distinct from the postmenopausal model previously used, in which tumor vessel density was increased in obese mice that were both ovariectomized and aged [34]. In conjunction with another report showing a lack of difference in tumoral vessel density between lean and obese mice [52], the observations made suggest that the association between tumoral vessel density with the presence of obesity is both age- and model-dependent.

In clinical studies, increased cytotoxic lymphocytes within tumors predict improved patient outcome [53]. Apart from inducing tumor cell death, cytotoxic lymphocytes may also induce G_1_-cell cycle arrest [54], which can stain positive for Ki67, albeit at a lower intensity, as Ki67 is present at all phases of the cell cycle except G_0_ [55]. This mechanism potentially may explain the slower tumor growth observed in the BP3KO mice despite similar Ki67^+^ and cleaved caspase-3^+^ cell indexes. Notably, the number of infiltrating T-cells was greater in tumors implanted in the BP3KO mice, regardless of diet. Further, the surprising observation that the normal positive relationship between tumor growth and vessel density, seen in wild-type mice, was reversed in BP3KO mice is consistent with a possible role for higher vessel density in facilitating T-cell infiltration [56].

This study has revealed that IGFBP-3 is integrally involved in the mechanism underlying the enhanced response of mammary tumors to an obese environment. We speculate that IGFBP-3 acts, at least in part, to protect against the potential growth-inhibitory effect of tumor T-cells, and may modulate the relationship between tumor vascularity and T-cell infiltration. We have shown that the growth-stimulatory effect of obesity is enhanced in the absence of host-derived IGFBP-3, where high-fat feeding causes increased tumor growth relative to chow diet. This suggests that the tumor growth-suppressive environment created by low host IGFBP-3 can, in part, be negated with development of obesity. In conclusion, our results indicate that IGFBP-3 has a potentially stimulatory role in mammary tumor growth *in vivo* and may contribute to mammary tumor progression in the context of obesity. This suggests that targeting IGFBP-3-dependent signaling pathways may be an effective approach to treating breast cancer in an obese environment.

## MATERIALS AND METHODS

### Mice and tumor cells

Animal studies were approved by the institutional Animal Care and Ethics Committee (Protocol 1305-003A). IGFBP-3 global knock-out (BP3KO) mice, with a disruption in exon 1 including the IGFBP-3 translation start site and the signal peptide, were generated and bred onto a C57BL/6 background [57] and a colony established at the Kolling Institute.

Six-week-old female BP3KO and wild-type (WT) C57BL/6 mice were fed either control chow diet (6% fat, 23% protein; Gordon's Specialty Feeds, NSW, Australia) or high-fat diet (47.7% fat, 19.5% protein; prepared in-house), for 15 weeks. Food intake per mouse was estimated by the weight of food dispensed and remaining in each cage every 3 days. The mice were group-housed to a maximum of 5 mice per cage. After a 15-week feeding period, mice were injected with EO771 mouse mammary tumor cells, provided by Prof. Robin Anderson, Peter MacCallum Cancer Centre, Melbourne, Australia. These cells, maintained in RPMI 1640 medium supplemented with 5% fetal calf serum and harvested at passages 12-13 for injection, have undetectable IGFBP-3 mRNA or protein. Cells (5 × 10^5^ in 80 μL serum-free RPMI medium mixed with 40 μL growth factor-free Matrigel (BD Biosciences, Franklin Lakes, NJ)) were injected into the left fourth mammary gland. Palpable tumors were measured with calipers. Mice were sacrificed when the largest tumor reached 1000 mm^3^ (calculated as ^1^/_2_ × *L* × *W^2^*). Mice were fasted overnight before cardiac puncture under anesthesia to collect blood. Tumors, mammary and visceral adipose tissue were weighed, sectioned and either snap-frozen in liquid nitrogen or placed into 10% neutral buffered formalin (POCD Scientific, Artarmon, NSW, Australia).

### Gene expression analysis

Total RNA from adipose tissue and tumors was extracted with TRIzol (ThermoFisher, Scoresby, VIC, Australia) and purified with Direct-zol RNA MiniPrep (Zymo Research, Irvine, CA). RNA was quantitated using a Nanodrop ND-1000 Spectrophotometer (NanoDrop Technologies, Wilmington, DE) and reverse-transcribed with Maxima H Minus Reverse Transcriptase (ThermoFisher) according to the manufacturer's instructions. Quantitative real-time PCR was performed in an ABI 7900HT (Applied Biosystems, Foster City, CA) using Taqman probes (Applied Biosystems) for murine PAI-1 (*Serpine1*; Mm00435857_m1), Fabp4 (*Fabp4*; Mn00445878_m1), adiponectin (*Adipoq*; Mm00456425_m1), Pref-1 (*Dlk1*; Mm00494477_m1), Emr1 (*Adgre1*; Mm00802529_m1), Pparg (*Pparg*; Mm00440940_m1) and MCP-1 (*Ccl2;* Mm00441241_m1). Transcripts for each sample were quantitated in triplicate, relative to importin-8 (*Ipo8*; Mm01255158_m1) for adipose tissue samples [58] or hydroxymethylbilane synthase (*Hmbs*; Mm01143545) for tumor samples.

### Cytokine measurement

Fasting tail-vein glucose was measured by glucometer (Nipro Diagnostics, Fort Lauderdale, FL) just before sacrifice. Serum insulin levels were measured using the Mouse/Rat Insulin ELISA kit (RayBiotech, Norcross, GA). PAI-1, leptin, resistin and MCP-1 were measured in serum by Luminex xMAP using a Milliplex MAP Mouse Adipokine Multiplex Assay (MADKMAG-71K, Millipore, Billerica, MA). IGFBP-3 was measured by mouse IGFBP-3 ELISA (R&D Systems, Minneapolis, MN). IGF-1 was measured by in-house radioimmunoassay.

### Immunohistochemical analysis

Tissues were fixed in formalin for 24 hours and paraffin-embedded. Four micron sections were deparaffinized and antigen retrieval was performed with either citrate buffer, pH 6 (for Ki67) or Tris-EDTA buffer, pH 9 (for caspase-3, CD3 and CD31). Sections were boiled in retrieval buffer for 20 minutes, then cooled for 20 minutes. Using an automated slide stainer (Dako Australia, North Sydney, NSW, Australia) sections were quenched with hydrogen peroxide, incubated with primary antibody then secondary antibody (K4003, Dako) and revealed with diaminobenzidine (Dako) or ImmPact NovaRed Peroxidase Substrate (Vector Laboratories, Burlingame, CA). Sections were counterstained in Mayer's hematoxylin. Primary antibodies were: Ki67 (ab66155, Abcam, final dilution 1:250), cleaved caspase-3 (CST9661, Cell Signaling Technology, Beverly, MA, final dilution 1:300), CD3 (A0452, Dako, final dilution 1:400) and CD31 (ab28364, Abcam, final dilution 1:100). Images were analyzed using CellProfiler, Broad Institute, MA [59], quantifying the number of positively stained pixels or total number of cells, identified by hematoxylin staining and the number of diaminobenzidine- or ImmPact NovaRed-positive cells present in the images. See Supplementary Methods for further details.

### Histological examination

Four-micron sections were cut from formalin-fixed paraffin-embedded omental and mammary fat pads, stained with hematoxylin and eosin and imaged. Eight representative fields of view were analyzed using Adiposoft software [60] for adipocyte size.

### Statistical analysis

Statistical significance was determined by 2-way ANOVA followed by post-hoc Tukey's test, repeated measures 2-way ANOVA (for tumor volume changes), *t*-test or by Spearman's correlation test, as indicated. GraphPadPrism v.6.00 (GraphPad Software, La Jolla, CA) was used for all analyses except for the repeated measures 2-way ANOVA which was analyzed using SPSS Statistics v.22 (IBM, Armonk, NY).

## SUPPLEMENTARY MATERIALS FIGURES


