# Supplementary material for: Insulin-like growth factor binding protein-3 links obesity and breast cancer progression
Source: Oncotarget. 2016 Jul 18;7(34):55491–505. doi: 10.18632/oncotarget.10675 (PMC5342431; doi:10.18632/oncotarget.10675)
Supplement: Supplementary file 1 [file oncotarget-07-55491-s001.pdf]

Insulin-like growth factor binding protein-3 links obesity and breast cancer progression

SUPPLEMENTARY METHODS

Quantification of stained sections

Tumor sections were imaged using a Zeiss Axioscan slide-scanning microscope with a 10X objective (Plan-Apochromat 10X/0.4 M27) for Ki67, CD3, and cleaved caspase-3 and 20X objective (Plan-Apochromat 20X/0.45 M27) for CD31 and each field-of-view was saved as an 8-bit TIFF file. All of the images obtained were analyzed using CellProfiler software, Broad Institute, MA (27) where the total number of cells, identified by hematoxylin staining and the number of DAB- or ImmPact Red-positive cells present in the images was quantified. The number of antigen-positive cells is expressed as a percentage of the total number of cells identified in the images. The total number of field-of-view images analyzed per section ranged from 100 – 500, depending on the size of section and magnification used. The total cell number quantified across images was positively correlated with tumor weight ($r^2 = 0.599$).

Total vessel density in the tumors was quantified by dividing the sum of the total number of $CD31^+$ pixels against that of total $hematoxylin^+$ pixels present in each field of view taken.

CellProfiler analysis was set up for each antigen by selecting nine representative fields of view across sections to account for intra- and inter-tumoral heterogeneity and basing the thresholds for identification of these antigens on these fields of view. As an automated slide stainer was used to stain large batches of slides for each antigen, consistency of staining was maintained across sections.

SUPPLEMENTARY FIGURES

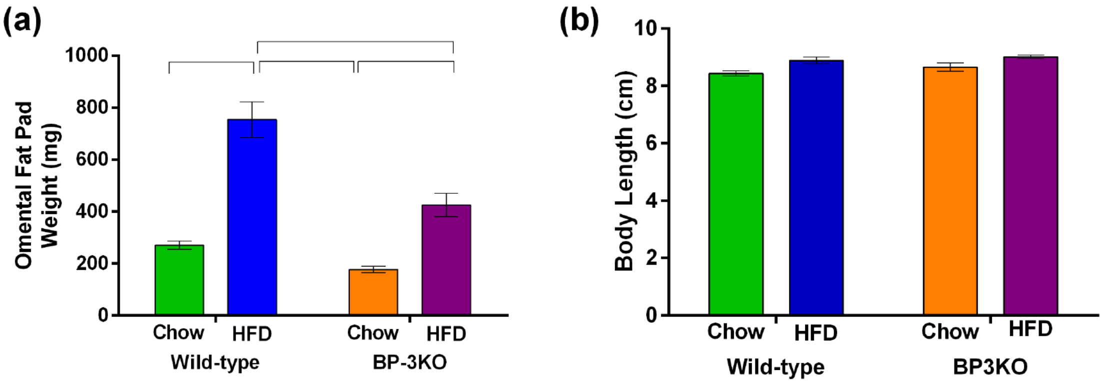

Supplementary Figure S1: a. Omental fat pad weights (p<0.0001 for genotype and diet by 2-way ANOVA, n = 22 - 36 per group). **b.** Comparison of body lengths of wild-type and BP3KO mice after 15 weeks of HFD (p < 0.002 for diet by 2-way ANOVA, n = 7 – 20 per group). Brackets show significant differences by *post-hoc* Tukey's test. Data are shown as means ± SEM.

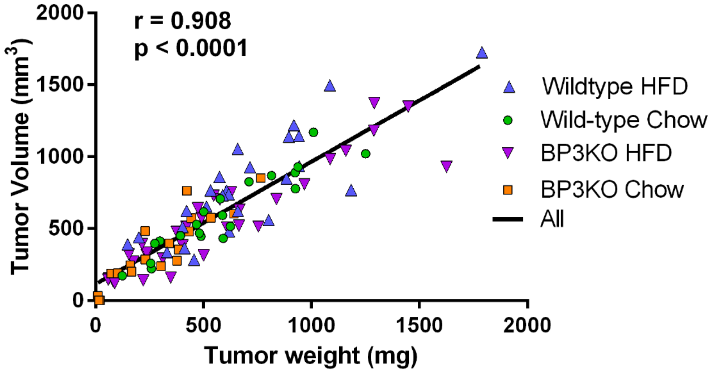

Supplementary Figure S2: Association between tumor volume and tumor weight (p < 0.0001, Spearman's correlation test).

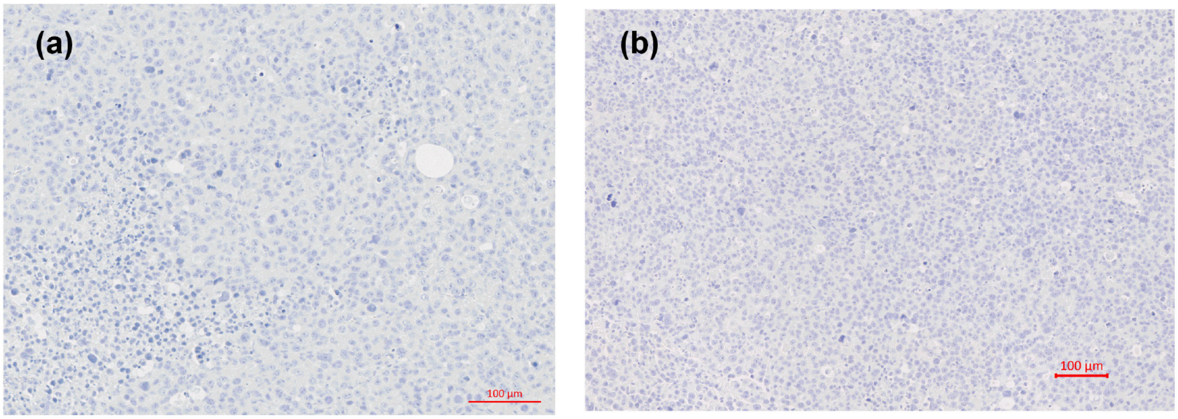

Supplementary Figure S3: Representative images of sections stained with the respective isotype controls for **a.** Ki67 and **b.** cleaved caspase-3.

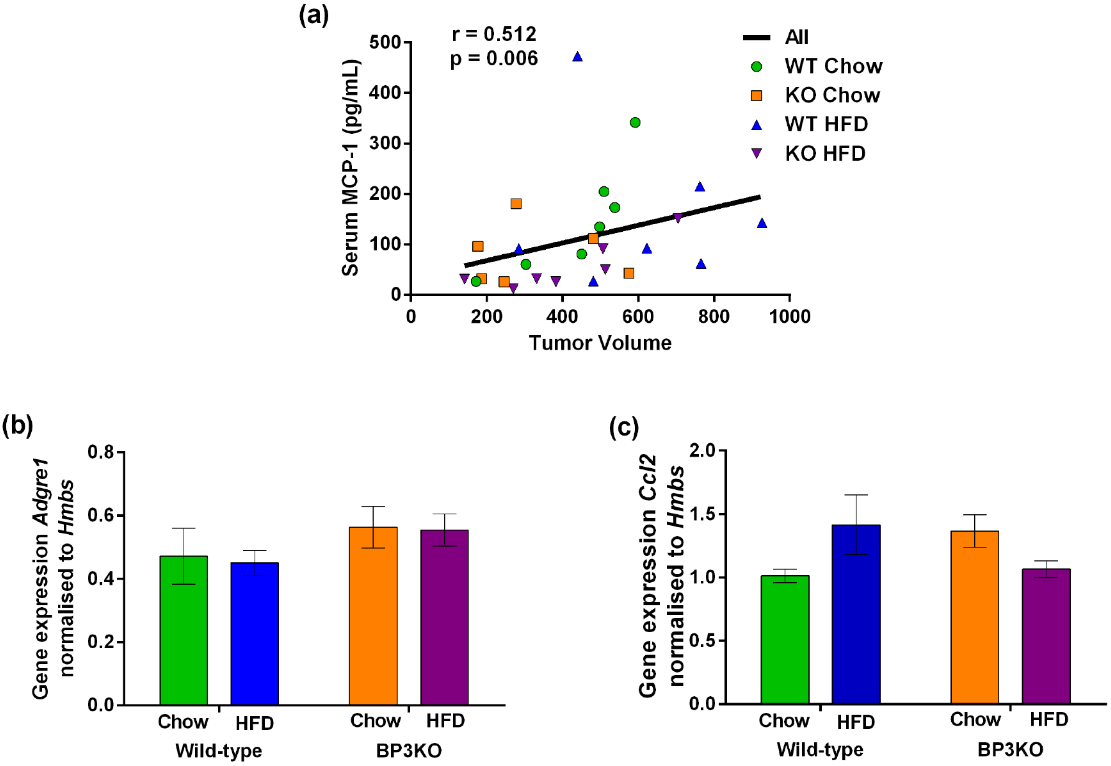

Supplementary Figure S4: Macrophage infiltration into tumors in wild-type and BP3KO mice on chow and HFD is similar despite differences in circulating MCP-1 levels. a. Serum MCP-1 levels are positively associated with tumor volume, p = 0.006, Spearman's correlation test. Tumor gene expression of **b.** macrophage marker *Adgre1* (emr-1), and **c.** *Ccl2* (mcp-1) is not different among groups by 2-way ANOVA, n = 8 per group. Data are mean values ± SEM.
